# 지역사회 거주 중년 이후 한국성인의 중성지방과 대장암 발생과의 관계 연구: 지역사회기반 코호트 이차 자료 분석

**DOI:** 10.7586/jkbns.25.084

**Published:** 2026-02-27

**Authors:** Bo-Kyoung Cha

**Affiliations:** Department of Nursing, Hanseo University, Seosan, Korea; 한서대학교 간호학과

**Keywords:** Cohort studies, Colorectal neoplasms, Diabetes mellitus, Hypertriglyceridemia, Triglycerides, 코호트 연구, 대장암, 당뇨병, 고중성지방혈증, 중성지방

## Abstract

**Purpose:**

This study examined the association between serum triglyceride levels and the incidence of colorectal cancer (CRC) among community-dwelling middle-aged and older adults.

**Methods:**

The study included 8,371 adults aged 40~69 years without CRC at baseline who were enrolled in the Korean Genome and Epidemiology Study and followed biennially for 20 years to ascertain incident CRC cases. Participants were classified into four groups according to baseline triglyceride quartiles (Q1 = 32~99 mg/dL, Q2 = 100~134 mg/dL, Q3 = 135~187 mg/dL, and Q4 = 188~498 mg/dL). Kaplan–Meier survival analysis with the log-rank test and Cox proportional hazards regression models were used to evaluate CRC incidence across quartiles.

**Results:**

During the follow-up period, 104 participants (1.24%) developed CRC. The overall incidence rate of CRC was 0.78 per 1,000 person-years, with rates of 0.95, 0.48, 0.70, and 1.01 per 1,000 person-years in Q1, Q2, Q3, and Q4, respectively. CRC-free survival did not differ significantly among the quartile groups (log-rank χ^2^ = 7.55, *p* = .056). However, after adjustment for major confounding factors, participants in both Q1 and Q4 exhibited a significantly higher risk of CRC compared with those in Q2 (Q1: hazard ratio [HR] = 2.13, 95% confidence interval [CI] = 1.15~3.95, p = .016; Q4: HR = 1.95, 95% CI = 1.02~3.73, *p* = .043).

**Conclusion:**

Serum triglyceride levels were associated with CRC risk in this population. Compared with Q2, CRC risk was elevated among those in both Q1 and Q4, indicating a non-linear relationship between triglyceride levels and CRC incidence. These findings highlight the potential value of preventive strategies tailored to serum triglyceride profiles, including identifying risk groups and improving CRC screening adherence.

## 서론

### 1. 연구의 필요성

국제 암 통계에 따르면[1], 2022년 우리나라에서 대장암은 전체 암의 12.4%를 차지해 두 번째로 흔한 암으로, 남녀 모두에서 높은 발생 부담을 보인다. 대장암 환자의 5년 상대 생존율은 2000년 초반 67.0%에서 최근 74.6%로 꾸준히 향상되었으나[2], 여전히 전체 암 중 사망 원인 3위를 차지하고 있으며[1], 수술 및 항암화학요법 등의 치료 과정에서 겪는 신체적·정신적·사회적 어려움으로 삶의 질 저하가 보고되고 있다[3,4]. 이러한 점은 대장암 발생과 사망, 삶의 질 측면에서 복합적인 부담이 존재함을 보여주며, 치료 중심 접근만으로는 효과적인 대장암 관리에 한계가 있음을 시사한다. 암을 체계적으로 관리하기 위해 암 예방, 조기검진, 진단, 치료, 회복·생존자 관리 등 암의 여정(cancer journey)에 따른 포괄적 접근이 요구되므로[5], 대장암 발생에 영향을 미치는 위험 요인을 탐색하고 이를 기반으로 한 예방 전략의 확대가 필요하다.

대장암은 생활습관 요인뿐 아니라 비만, 비정상적 혈당 상승, 이상지질혈증 등 대사 요인과 연관성이 높으며[6-13], 이 가운데 중성지방은 만성염증, 인슐린 저항성, 산화적 스트레스 증가 등 대장암 발암과정과 관련된 주요 기전과 연관된 것으로 보고되면서[10,14-17], 대장암 발생의 잠재적 표지자로서 관심이 증가하고 있다[9,10].

중성지방과 대장암 발생의 연관성에 대한 연구는 주로 대규모 국가 기반 코호트가 형성된 국외 연구에서 검토되었으나, 일부 연구에서는 유의한 연관성이 관찰되지 않거나[18] 대장암 부위에 따라 상이한 결과를 보이는 등[19] 개별 연구 수준에서는 결과가 일관적이지 않았다. 이러한 불일치는 연구 대상 인종, 지역적 특성, 조정된 공변량 및 추적기간 차이 등 방법론적 다양성에서 기인한 것으로 보고된 바 있다[9]. 그럼에도 불구하고 최근 메타분석 연구에서는 중성지방과 대장암 발생 위험 간의 연관성이 통계적으로 유의한 것으로 제시되었으며[9], 대규모 코호트를 활용한 실증적 연구에서도 두 변수의 관련성이 점차 축적되고 있다[7,20,21]. 국내에서도 대규모 코호트의 구축에 따라 중성지방과 대장암 발생과의 관계를 전향적으로 검증한 연구들이 보고되고 있으나, 이들 연구들은 연구 대상의 연령 범위나 모집단의 특성에 따라 결과 해석 범위에 일정한 제한을 가진다. Chang 등[20]의 고중성지방혈증의 지속성과 조기 발병 대장암 연구는 조기 발병 대장암을 만 50세 이전에 진단된 경우로 정의하고 20~49세 성인을 대상으로 분석하였다는 점에서, 대장암 발생이 뚜렷하게 증가하는 50세 이후 연령층[2]에게 연구 결과를 적용하는 데는 어려움이 있다. 또한, Min 등[21]은 의료 접근성이 높은 도시지역 의료기관 검진센터에 내원한 40세 이상 성인을 대상으로 모집한 도시기반 코호트를 이용하였으므로, 연구결과를 생활환경이 상이한 인구집단으로 확장하여 해석하는 데에는 신중한 접근이 요구된다. 선행연구에서 도시, 지역사회, 농촌의 이상지질혈증 유병률은 각각 34.5%, 47.3%, 53.3%였으며, 대사증후군 유병률도 25.2%, 32.7%, 44.3%로 지역에 따라 뚜렷한 차이를 보였다는 점은[22], 국내 일반 인구집단 내에서도 거주 지역에 따른 대사 특성의 이질성이 존재함을 시사한다. 이러한 점을 고려할 때, 우리나라 국민의 중성지방과 대장암 발생 위험 간의 연관성을 보다 폭넓은 인구집단 맥락에서 이해하기 위해 대장암 발생 위험이 높은 연령층을 포함하여 생활환경의 다양성을 반영한 연구가 요구된다.

한편, 대장암 발생 위험에는 연령 증가, 남성, 현재 흡연, 현재 음주, 낮은 신체활동량, 고지방 섭취 등이 관여하는 것으로 보고되고 있고, 비만, 당뇨병, 인슐린저항성 증가, 총콜레스테롤 상승, 고밀도 지단백콜레스테롤 감소 역시 주요 위험요인으로 제시되었다[7,9,14,20,21,23]. 따라서 중성지방이 대장암 발생에 미치는 독립적인 영향을 평가하기 위해서는 이러한 요인을 통제요인으로 고려한 분석이 필요하다.

이를 종합해보면, 최근 국내외 연구는 혈중 중성지방이 대장암 발생 위험과 유의하게 연관된다는 근거를 제시하고 있으나, 이러한 결과를 다양한 연령층과 지역사회 인구집단으로 확장하여 검증할 필요성이 제기된다. 특히, 대장암 발생률이 증가하는 40세 이후 연령층[2]을 포함하고, 유사한 생활환경에서 생활하며 추적의 안정성이 확보된 모집단 대상의 장기간 추적자료를 활용한 연구가 필요하다고 판단된다.

한국인 유전체 역학조사사업 지역사회 기반 코호트(Korean Genome and Epidemiology Study–Ansan and Ansung study, KoGES-Ansan and Ansung study)는 중소도시와 농촌 지역 주민을 대상으로 한 20년 장기 추적 코호트로, 지역사회 거주 성인의 생활습관과 대사 특성에 따른 질병 발생을 관찰할 수 있는 자료를 제공하고 있다[24]. 이에 본 연구는 안산·안성 코호트 자료를 활용하여 혈중 중성지방과 대장암 발생 위험 간의 연관성을 확인하고, 생활습관 및 대사 요인을 통제한 상태에서도 중성지방이 독립적인 위험요인으로 작용하는지를 평가하고자 하였다. 본 연구 결과는 대장암 발생에 대한 중성지방의 잠재적 역할을 제시함으로써 향후 대장암 예방전략 수립을 위한 근거 자료로 활용될 수 있을 것이다.

### 2. 연구의 목적

본 연구는 우리나라 지역사회에 거주하는 중년이후 성인의 혈중 중성지방과 대장암 발생 위험의 관계를 규명하기 위하여 수행되었으며, 구체적 목적은 다음과 같다.

• 인구학적 특성 및 건강관련 특성과 중성지방 사분위 집단과의 연관성을 확인한다.

• 인구학적 특성 및 건강관련 특성과 대장암 발생 위험과의 연관성을 확인한다.

• 중성지방 사분위 집단에 따른 1,000 인년(person-years)당 대장암 발생률을 산출하고 생존곡선의 차이를 확인한다.

• 대장암 발생 위험이 가장 낮은 집단을 기준으로 중성지방 사분위 집단 별 대장암 발생 위험의 차이를 비교한다.

## 연구 방법

### 1. 연구 설계

본 연구는 한국인 유전체 역학조사사업 지역사회 기반 코호트의 20년 추적자료를 이용하여, 지역사회에 거주하는 40~69세 성인의 혈중 중성지방과 대장암 발생 위험의 연관성을 확인한 이차 자료 분석 연구이다.

### 2. 연구 대상

지역사회기반 코호트 사업에서 기반조사(baseline)는 2001~2002년에 안산과 안성에 거주하는 40~69 세 남녀 10,030명(안산: 5,012명, 안성: 5,018명)을 대상으로 진행되었다. 이후 2년마다 추적조사가 이루어져, 추적조사 참여자는 1차 8,603명, 2차 7,515명, 3차 6,688명, 4차 6,665명, 5차 6,238명, 6차 5,906명, 7차 6,318명, 8차 6,157명, 9차 5,854명, 10차 5,5511명이었다[24].

본 연구의 대상자 선정기준은 기반조사에서 10개 악성종양(폐암, 위암, 간암, 대장암, 췌장암, 자궁암, 유방암, 담낭암, 갑상선암, 전립선암)의 진단을 받지 않은 자, 기반조사에서 지질강하제를 복용하지 않는 자, 기반조사에서 중성지방이 500 mg/dL미만인 자, 기반조사 시점으로부터 1년이 경과한 이후 대장암을 진단받은 자, 추적조사에서 대장암 진단 여부를 최소 1회 이상 응답한 자로 하였다. 대장암 발생자의 추적관찰기간은 기반조사일부터 대장암이 처음 진단된 추적조사의 조사일까지로 정의하였고, 대장암이 발생하지 않은 대상자의 추적관찰기간은 기반조사일부터 10차 추적조사 중 마지막으로 참여한 차수의 조사일까지로 정의하였다.

**Figure 1.**
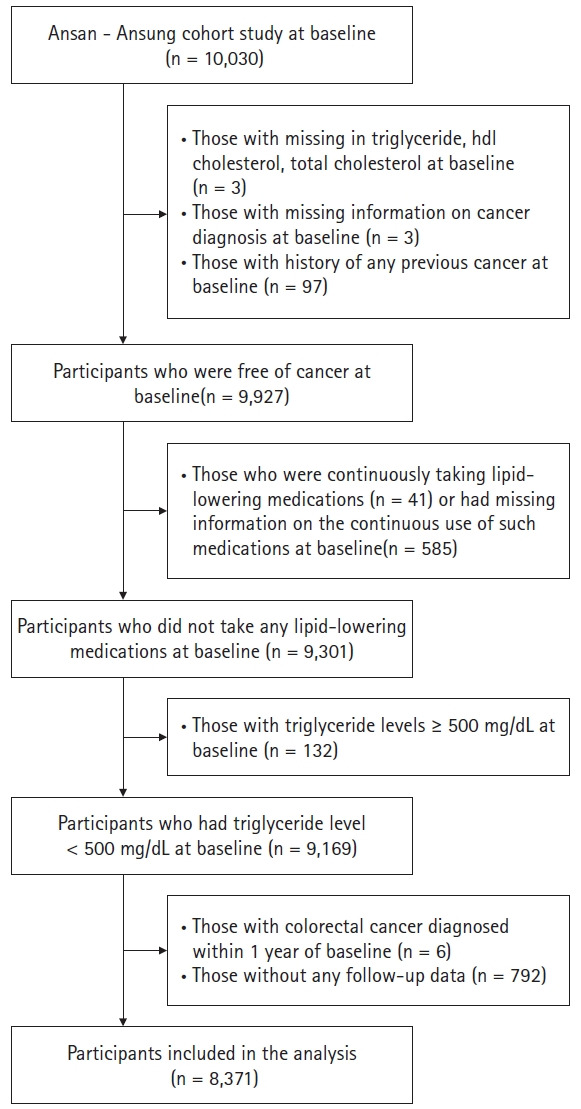
Flowchart of study subjects’ selection.

연구대상자 선정기준에 따라 기반조사에 참여한 10,030명 중 다음과 같은 단계로 연구대상자를 제외하였다(Figure 1). 1) 기반조사에서 중성지방, 고밀도지단백콜레스테롤, 총콜레스테롤에 결측 치가 있는 자(n = 3), 기반조사에서 10개 악성종양 진단 여부에 무응답하였거나(n = 3) 악성종양 진단을 받은 자(n = 97), 2) 기반조사에서 지질강하제 복용 지속 여부에 무응답하였거나(n = 585) 지질강하제를 지속적으로 복용하고 있는 자(n = 41), 3) 기반조사에서 중성지방이 500 mg/dL 이상인 자(n = 132), 4) 기반조사 이후 1년 이내 대장암을 진단받은 자(n = 6), 추적조사에 전혀 참여하지 않았거나 대장암 진단 여부에 한차례도 응답하지 않은 자(n = 792)의 순서로 제외하였다. 이 과정을 거쳐 최종 분석에 포함된 대상자는 8,371명이었다. 기반조사에서 악성암으로 인해 제외된 97명은 자궁암 31명, 위암 28명, 유방암 17명, 대장암 11명, 폐암 6명, 췌장암 2명, 간암 1명, 담낭암 1명이었다.

### 3. 연구 도구

#### 1) 대장암

대장암은 의사로부터 대장암(악성종양)으로 진단받은 경우로 정의하였으며, 자가보고 방식으로 조사하였다. 기반조사에서는 “일생 동안 귀하는 병의원에서 의사로부터 아래의 질병으로 진단받은 적이 있습니까?” 라는 질문 안에 “대장암 진단여부” 항목이 포함되었고, 추적조사에서는 “지난 2년간(지난 코호트 검진 이후) 귀하는 병의원에서 의사로부터 아래의 질병으로 진단받은 적이 있습니까?” 라는 질문 하에 “대장암 진단여부” 항목이 포함되어 있다[25].

#### 2) 중성지방

중성지방은 기반조사에서 8시간 이상 금식 후 채혈하여 측정한 원측정값과 변환값이 있으며, 본 연구에서는 변환값을 사용하였다. 중성지방 변환값은 검사 장비 변경으로 인한 오차를 최소화하기 위해 검진일에 따라 기준장비 전환식[중성지방 변환값 = 21.073 + (0.942 × 중성지방 원측정값)]을 도출하여 산출한 값을 의미한다[25]. 중성지방 500 mg/dL 이상은 만성콩팥병, 갑상선기능저하증 등과 같은 이차적 원인이나 유전적 요인의 가능성이 있어 연구대상에서 제외하였다[26]. 중성지방 분포에 따라 연구대상자를 사분위수(quartile, Q) 기준의 4개 집단(Q1~Q4)으로 분류하였으며, 각 집단의 중성지방 범위는 Q1: 32~99 mg/dL, Q2: 100~134 mg/dL, Q3: 135~187 mg/dL, Q4: 188~498 mg/dL였다.

#### 3) 위험요인

위험요인은 기반조사 시점의 연령, 성별, 음주상태, 흡연상태, 총신체활동량, 영양소 섭취량, 체질량지수, 2형 당뇨병, 고혈압, 인슐린저항도, 고밀도지단백콜레스테롤, 총콜레스테롤을 포함하였다. 음주상태는 비음주, 과거음주, 현재음주로 분류하였으며, 흡연상태는 비흡연, 과거흡연, 현재흡연으로 구분하였다[27]. 총신체활동량은 육체적 활동과 운동 문항에서 산출한 당대사량(metabolic equivalent of task)과 활동 시간을 이용하여 계산한 값을 적용하였다[25]. 영양소 섭취량은 개인이 하루동안 섭취한 모든 음식 및 식품으로부터의 섭취량으로, 탄수화물 에너지 섭취비율(%kcal), 지방 에너지 섭취비율(%kcal), 식이섬유 섭취량(g)으로 정의하였다. 탄수화물 에너지 섭취비율과 지방 에너지 섭취비율은 해당 영양소 섭취량과 총섭취에너지를 이용하여 산출하였다. 탄수화물 에너지 섭취비율은 적정비율(55%~65%)을 기준으로 < 55%, 55%~65%, > 65%로 분류하였으며, 지방 에너지 섭취비율은 적정비율(15%~30%)을 기준으로 < 15%, 15%~30%, > 30%로 분류하였다[28]. 식이섬유 섭취량(g)은 사분위수에 따라 4개 집단 (Q1~Q4)으로 구분하였다. 체질량지수는 체중(kg)/[신장(m)]^2^로 산출하였다. 한국비만학회의 기준[29]에 따라 저체중 < 18.5 kg/m^2^, 정상체중 18.5~22.9 kg/m^2^, 과체중 23~24.9 kg/m^2^, 비만 ≥ 25 kg/m^2^으로 구분한 후, 저체중과 정상체중을 통합하여 ≤ 22.9 kg/m^2^, 23~24.9 kg/m^2^, ≥ 25 kg/m^2^의 세집단으로 분류하였다. 2형 당뇨병은 8시간 이상 금식 후 (1) 공복혈장혈당 ≥ 126 mg/dL, (2) 75 g 경구 포도당 부하검사 후 2시간 혈장혈당 ≥ 200 mg/dL, (3) 당화혈색소 ≥ 6.5% 중 하나 이상에 해당되거나 경구혈당강하제나 인슐린을 투여하는 경우로 정의하였다[30]. 고혈압은 수축기혈압 140 mmHg 이상이거나 이완기혈압 90 mmHg 이상, 또는 항고혈압제를 복용하는 경우로 정의하였다[31]. 혈압은 양쪽 팔에서 각각 2회 측정하였다. 수축기혈압 평균이 더 높은 팔의 값을 사용하였으며, 양쪽 팔의 수축기혈압 평균이 동일한 경우에는 이완기혈압의 양측 평균값을 적용하였다. 인슐린저항도는 Homeostatic Model Assessment for Insulin Resistance Index (HOMA-IR) 지수를 이용하여 공복혈장인슐린(µIU/mL) × 공복혈장혈당(mg/dL)/405 [32]로 산출하였다. HOMA-IR 수치가 2.0 이상에서 인슐린저항성과 관련된 대사이상 위험이 점차 증가하며, 2.2~2.5범위에서 위험성이 뚜렷하게 증가하였다는 보고를 근거로 2 미만, 2 이상 2.5 미만, 2.5 이상의 세집단으로 분류하였다[33,34]. 고밀도지단백콜레스테롤과 총콜레스테롤은 8시간 이상 금식 후 채혈하여 측정한 원측정값에 검사장비 변경에 따른 오차를 최소화하기 위하여 전환식을 적용하여 산출한 변환값을 사용하였으며[25], 사분위수를 기준으로 4개 집단(Q1~Q4)으로 분류하였다.

### 4. 자료 수집

본 연구의 자료는 보건의료연구 자원정보센터(Clinical & Omics Data Archive)로부터 지역사회기반 코호트 반복추적조사 통합자료(KoGES follow up study combined data)를 분양 받아 분석에 이용하였다. 연구자는 보건의료연구 자원정보센터를 통해 연구계획서, 생명윤리심의위원회 (Institutional Review Board, IRB) 승인서, 분양 서약서, 개인정보 수집·이용 동의서, 인체자원정보 이용계획서 등 분양심의를 위해 필요한 서류를 제출하고 데이터 분양 승인을 받았다. 승인된 원시 자료는 보건의료빅데이터 공유·활용 플랫폼(Healthcare big data sharing and utilization platform)에서 이용할 수 있다. 원시자료는 대상자를 확인할 수 없도록 익명화 처리되어 있으며, 승인받지 않은 모든 분석 자료는 플랫폼에서 반출이 불가능하다.

### 5. 자료 분석

통계 분석은 보건의료 빅데이터 공유·활용 플랫폼의Jupyter Notebook 환경에서 R for Windows (version 4.3.0; R Foundation for Statistical Computing, Vienna, Austria) [35]을 이용하여 수행하였다. 기술통계 및 생존분석(survival analysis)을 실시하였으며, 구체적인 분석 방법은 다음과 같다. 중성지방 사분위 집단별 인구학적 특성 및 건강 관련 특성에 대해 빈도, 백분율을 산출하였고, 이들 특성과 중성지방 사분위 집단의 연관성은 카이제곱검정(χ^2^ test)을 이용하여 분석하였다. 인구학적 특성 및 건강관련 특성과 대장암 발생 위험과의 관계는 단변량 콕스 비례위험 회귀모형(Cox proportional hazards regression model)으로 분석하였다. 중성지방 사분위 집단별 1,000 인년당 대장암 발생률을 산출하고, 카플란–마이어(Kaplan–Meier) 방법을 이용하여 중성지방 사분위 집단별 생존곡선을 추정하고 로그순위검정(log-rank test)으로 생존곡선간 차이를 비교하였다. 콕스 비례위험 회귀모형의 가정 충족 여부는 Kaplan–Meier 생존곡선의 형태와 숀펠드 잔차(Schoenfeld residuals)를 통해 종합적으로 평가하였다. 중성지방 사분위 집단별 대장암 발생 위험의 차이는 콕스 비례위험 회귀모형을 이용하여 위험비(hazard ratio, HR)와 95% 신뢰구간(confidence interval, CI)을 산출하여 분석하였다. 공변량은 단계적으로 보정하였으며, 모형1 (Model I)은 연령과 성별을, 모형2 (Model II)에는 선행연구에서 보고된 대장암 위험요인을 근거로 본 연구에서 중성지방 사분위 집단과의 연관성 분석 또는 단변량 콕스 비례위험 회귀모형에서 유의한 변수를 포함하였다. 회귀모형 분석 전 다중공선성과 비례위험가정에 위배되는 변수는 최종모형에서 제외하였다. ChatGPT (GPT-5.1)은 자료분석 과정에서 R 코드 작성 및 문법 오류 수정에 활용되었다.

### 6. 윤리적 고려

본 연구는 한서대학교 생명윤리위원회의 연구심의면제(IRB No.: HS25-04-01) 확인을 받았다. KoGES 수행기관은 자료수집 당시 참여자를 모집하여 참여 설명서를 이용하여 사업 목적을 충분히 설명하였다. 자발적 동의에 따르며 본인이 원하지 않으면 언제든지 사업 참여를 거절할 수 있으며, 이에 따른 어떠한 불이익도 없음을 설명하여 자율적 참여와 자유로운 중단에 대한 윤리규정을 준수하였다. 설명을 듣고 스스로 참여의사를 밝힌 대상자에 한하여 동의서를 받은 후 설문조사와 검진을 통해 자료와 인체 자원을 수집하였다[27].

## 연구 결과

### 1. 인구학적 특성 및 건강관련 특성과 중성지방 사분위 집단과의 연관성

대상자의 인구학적 특성 및 건강관련 특성과 중성지방 사분위 집단과의 연관성은 Table 1과 같다. 연구대상자 8,371명의 연령은 40~49세 48.1%, 50~59세 26.1%, 60~69세 25.8%이었으며, 성별은 남성 47.2%, 여성 52.8%였다. 인구학적 특성에서 연령(χ^2^ = 106.11, *p* < .001), 성별(χ^2^ = 193.85, *p* < .001)은 중성지방 사분위 집단과 유의한 연관성이 있었다. 건강관련 특성에서 음주상태(χ^2^ = 50.51, *p* < .001), 흡연상태(χ^2^ = 216.35, *p* < .001), 총신체활동량(χ^2^ = 49.09, *p* < .001)은 중성지방 사분위 집단과 유의한 연관성이 있었다. 2형 당뇨병 유무(χ^2^ = 236.76, *p* < .001), 고혈압 유무(χ^2^ = 301.42, *p* <.001), 체질량지수(χ^2^ = 570.12, *p* < .001), 인슐린저항도(χ^2^ = 289.41, *p* < .001), 고밀도지단백콜레스테롤(χ^2^ = 1406.11, *p* < .001), 총콜레스테롤(χ^2^ = 438.17, *p* < .001)는 중성지방 사분위 집단과 유의한 연관성이 있었다. 탄수화물 에너지 섭취비율(χ^2^ = 6.63, *p* = .357), 지방 에너지 섭취비율(χ^2^ = 10.46, *p* = .107), 식이섬유 섭취량(χ^2^ = 15.67, *p* = .074)은 중성지방 사분위 집단과 유의한 연관성이 없었다.

### 2. 인구학적 특성 및 건강관련 특성과 대장암 발생 위험과의 연관성

인구학적 특성 및 건강관련 특성과 대장암 발생 위험과의 연관성을 분석한 결과는 Table 2와 같다. 성별에서는 남성이 여성보다 대장암 발생 위험이 높았으며(HR = 0.61, 95% CI = 0.42~0.91, *p* = .014), 흡연 상태에서는 과거 흡연자(HR = 1.89, 95% CI =1.15~3.11, *p* = .012)와 현재 흡연자(HR = 1.75, 95% CI = 1.12~2.74, *p* = .014)가 비흡연자에 비해 대장암 발생 위험이 유의하게 높았다. 2형 당뇨병이 있는 경우(HR = 1.81, 95% CI =1.10~2.97, *p* = .020) 없는 경우 보다 대장암 발생 위험이 높았고, 지방 에너지 섭취비율 > 30%은 < 15% 보다 대장암 발생 위험이 유의하게 높았다(HR = 4.11, 95% CI = 1.29~13.06, *p* = .016). 연령, 음주상태, 총신체활동량, 탄수화물 에너지 섭취비율, 식이섬유 섭취량, 고혈압 유무, 체질량지수, 인슐린저항도, 고밀도지단백콜레스테롤, 총콜레스테롤은 대장암 발생 위험과 유의한 연관성이 없었다.

### 3. 중성지방 사분위 집단에 따른 1,000인년당 대장암 발생률과 생존곡선의 차이

**Figure 2.**
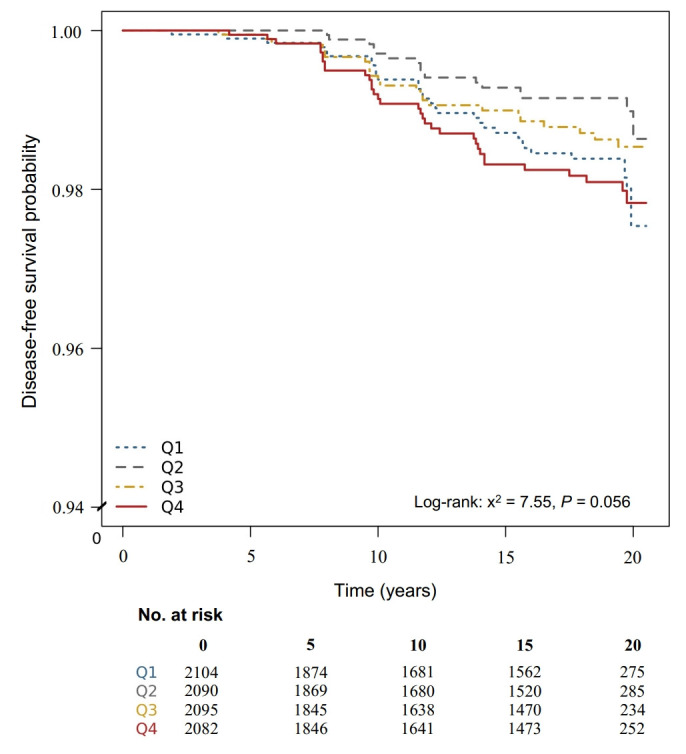
Kaplan–Meier curves for colorectal cancer–free survival by triglyceride quartiles.

전체 8,371명 중 104명에서 대장암이 발생하여 누적 발생율은 1.24%이었다. 추적기간 중앙값은 19.50년이었으며, 총 추적기간은 132,624년이었다. 이 기간동안 전체 대장암 발생률은 1,000인년당 0.78건이었으며, 중성지방 사분위 집단별 발생률은 각각 1,000인년당 Q1 0.95건, Q2 0.48건, Q3 0.70건, Q4 1.01건이었다(Table 3). 중성지방 사분위 집단별 대장암 발생위험에 대한 생존곡선(Figure 2)은 로그순위 검정에서 통계적으로 유의한 차이를 보이지 않았다(χ^2^ = 7.55, df = 3, *p* = .056).

### 4. 중성지방 사분위 집단에 따른 대장암 발생 위험

다변량 콕스 비례위험 회귀모형(모형2)에서는 최종 보정변수로 연령, 성별, 음주상태, 흡연상태, 총신체활동량, 체질량지수, 지방 에너지 섭취비율, 2형 당뇨병 유무, 고혈압 유무, 인슐린저항도, 고밀도지단백콜레스테롤, 총콜레스테롤을 포함하였다. 분석에 앞서 각 변수의 분산팽창지수(variance inflation factor)는 최대 2.68, 최소 1.01 로 다중공선성의 문제는 없었다. 또한, Schoenfeld 잔차 검정으로 비례위험 회귀모형의 가정 충족 여부를 확인한 결과, 비례위험 가정을 위배한 식이섬유 섭취량은 최종 회귀모형에서 제외하였으며, 회귀모형에 포함된 다른 모든 변수의 p값이 0.05 보다 커 비례위험가정은 충족된 것으로 판단하였다[36]. 중성지방 사분위 집단별 대장암 발생 위험은 대장암 발생률이 가장 낮은 중성지방 Q2 집단을 기준(reference)으로 분석하였다(Table 3). 보정 전 모형(crude model)에서는 Q2 집단에 비해 Q1 집단의 대장암 발생 위험비는 1.97배(95% CI: 1.08~3.60, *p* = .026), Q4 집단의 대장암 발생 위험비는 2.13배(95% CI: 1.17~3.87, *p* = .013) 유의하게 높았다. 연령 및 성별을 보정한 모형1 에서도 Q2 집단에 비해 Q1 집단의 대장암 발생 위험비는 2.12배(95% CI: 1.16~3.87, *p* = .014), Q4 집단의 위험비는 1.96배(95% CI: 1.08~3.56, *p* = .028) 유의하게 더 높았다. 공변수를 보정한 모형2에서도 Q2 집단에 비해 Q1 집단의 대장암 발생 위험비는 2.13배(95% CI: 1.15~3.95, *p* = .016), Q4 집단의 대장암 발생 위험비는 1.95배(95% CI: 1.02~3.73, *p* = .043) 유의하게 더 높았다. 중성지방 사분위 집단에 따른 대장암 발생 위험은 사분위가 증가함에 따라 점진적으로 증가하는 선형적 형태가 아니라, 중간 수준(Q2)을 기준으로 가장 낮은 집단(Q1)과 가장 높은 집단 (Q4)에서 대장암 발생 위험이 상대적으로 높게 나타나는 비선형적 패턴이 관찰되었다.

## 논의

본 연구는 중성지방이 대장암 발생 위험에 연관된 요인으로 관심이 집중되는 가운데, 지역사회기반 코호트의 20년간의 추적자료를 활용하여, 지역사회에 거주하는 40~69세 성인의 중성지방 사분위 집단에 따른 대장암 발생률을 확인하고, 혈중 중성지방이 대장암 발생 위험 요소로 영향을 미치는지를 파악하고자 시도되었다.

본 연구 대상자의 중성지방 사분위수 경계값은 99 mg/dL, 134 mg/dL, 187 mg/dL이었으며, Min등[21]의 연구에서 보고된 71 mg/dL, 102 mg/dL, 150 mg/dL보다 전반적으로 높은 수준에서 형성되었다. 이러한 차이는 두 연구에서 활용한 코호트의 모집 방식과 모집단 특성의 차이에 기인했을 가능성이 있다. 본 연구는 농촌(안성)과 중소도시(안산) 지역 주민을 대상으로 확률 표출법을 적용해 표본을 추출한 지역사회기반 코호트를 이용한 반면[37], Min 등[21]의 연구는 도시지역 의료기관의 건강검진센터에 내원한 남녀 대상의 도시기반 코호트를 활용하였다[38]. 도시기반 코호트는 주로 대도시 소재의 건강검진센터 및 대형 종합병원 중심의 38개 참여기관에서 진행되어[38], 대상자 모집단계에서 검진기관 접근성, 검진 이행 수준 등의 특성이 작용했을 가능성이 있다. 실제로 우리나라에서는 40세 이상 성인을 대상으로 2년 주기의 국가건강검진 제도가 시행되고 있으나, 지역 시군구별 소득 수준이 높고 병원수가 많을수록 수검률이 높다는 보고가 있으며[39], 정기적으로 건강검진에 참여한 대상자는 비참여자에 비해 심혈관질환 관련 위험요인이 상대적으로 적은 것으로 나타난 바 있다[40]. 그러므로 두 코호트의 모집단 특성 차이는 대상자의 생활양식, 식이 및 음주 습관, 의료이용 환경과 건강 서비스 이용 수준의 차이로 이어질 수 있으며, 이러한 특성은 혈중 중성지방 수준과 밀접하게 관련되어 있어 결과적으로 중성지방 분포 차이에 영향을 미쳤을 가능성이 있다고 사료된다.

인구학적 특성 및 건강 관련 특성에 따라 중성지방 사분위 분포는 뚜렷한 차이를 보였다. 본 연구에서 50~59세 연령층과 남성에서 중성지방 Q4 집단에 속하는 비율이 가장 높았으며, 이러한 연령 및 성별 분포는 남성의 고중성지방혈증 유병률이 중년에서 정점을 보인다는 국내 연구와 유사한 양상이라 할 수 있다[41]. 중년 남성에서 중성지방 수치가 상대적으로 높게 나타나는 현상은 연령 증가에 따른 내장지방 축적과 함께 흡연, 과도한 음주, 신체활동 감소와 같은 생활습관 요인이 복합적으로 작용한 결과로 고려할 수 있다[41]. 또한, 본 연구에서 현재 흡연, 현재 음주, 체질량지수 ≥ 25 kg/m², 낮은 고밀도지단백콜레스테롤, 높은 총콜레스테롤, 2형 당뇨병 및 고혈압을 가진 집단에서 중성지방Q4 집단의 비율이 두드러지게 높았다. 이는 중성지방이 다양한 생활습관 및 대사 요인과 밀접하게 연관되어 있음을 보여주며[42], 대장암 발생 위험 분석에서 이러한 요인들을 공변량으로 고려해야 할 필요성을 뒷받침하는 결과라 사료된다.

생존곡선이 비교 집단 사이에 서로 교차하는 경우, 로그순위 검정은 집단간 차이를 충분히 반영하지 못할 수 있다는 한계가 보고된 바 있다[36,43]. 본 연구에서는 일부 중성지방 사분위 집단 간 생존곡선이 교차하는 양상이 관찰되었으며, 이러한 점을 고려하여 비례위험 가정 충족 여부를 확인한 후, 다변량 콕스 비례위험 회귀모형을 적용하였다. 특히 로그순위 검정은 일변량분석만 가능한 반면[36] 콕스 비례위험 회귀모형은 연령, 성별 및 위험요인을 보정하여 중성지방 사분위 집단 간의 독립적인 위험 차이를 추정할 수 있으므로, 보정 후 콕스 비례위험 회귀모형 결과를 중심으로 집단 간 위험 차이를 해석하였다. 분석 결과에서는 생활습관 요인과 대사요인을 통제한 후에도 혈중 중성지방은 지역사회에 거주하는 40~69세 성인의 대장암 발생 위험과 유의한 연관성을 보였다. 중성지방 증가는 세포에서 만성적으로 지속되는 경미한 염증 반응을 유발하고[10,14,16], 지방조직에서의 지방분해 및 간의 지방합성을 동시에 촉진하는 인슐린저항성의 증가[15-17]와 산화적 스트레스 활성 등[10,16,17]을 통해 대장암 발생과 관련된 기전을 강화하는 것으로 알려져 있다. 특히 지방조직에서 분비되는 염증성 사이토카인과 지방분해 과정에서 방출된 자유지방산은 발암 과정에 관여하는 요인으로 제시되어 왔다[10,14-16]. 이러한 기전은 중성지방 수치가 상대적으로 높은 집단에서 대장암 발생 위험이 증가하는 현상을 뒷받침한다. 실제로 본 연구에서도 중성지방 사분위 집단 중 1,000인년당 대장암 발생률이 가장 낮은 Q2 집단을 기준으로 비교했을 때, Q4 집단의 대장암 발생 위험은 1.95배 높게 나타났다. 코호트 연구를 통합한 메타분석에서는 중성지방이 높은 집단이 낮은 집단에 비해 대장암 발생 위험이 18% 증가한 것으로 보고되었으며[10], 대만의 대규모 코호트 연구에서도 중성지방 150 mg/dL 이상인 집단이 150 mg/dL 미만인 집단보다 대장암 발생 위험이 약 1.7배 높은 것으로 나타났다[7]. 또한 우리나라 도시 기반 코호트 연구[21]에서도 중성지방 200 mg/dL 이상인 집단에서 대장암 발생 위험이 약 1.26배 높았고, 사분위 분석 결과 Q1 집단에 비해 Q4 집단의 대장암 발생 위험이 1.42배 증가한 것으로 보고되었다. 이러한 선행연구들은 중성지방이 높은 집단에서 대장암 발생 위험이 증가하는 선형적 경향을 제시하고 있으며, 이는 본 연구에서 관찰된 Q4 집단의 위험 증가와 일관된 결과로 해석될 수 있다.

반면, 본 연구에서는 중성지방 Q2 집단보다 최하위 사분위 집단인 Q1에서 상대적으로 높은 대장암 발생 위험이 관찰되었으며, 이는 중성지방과 대장암 발생 위험 간의 관계가 비선형적 패턴일 가능성을 시사한다. 이러한 결과는 중성지방과 대장암발생 위험 간의 관계가 단순한 선형 관계가 아닐 수 있음을 보여준다는 점에서 기존 연구와 차별성을 보인다. 이러한 패턴은 심부전 환자를 대상으로 한 대규모 코호트 연구에서도 관찰되었는데, 중성지방 수치가 중간 수준보다 낮은 경우 심부전으로 인한 입원 및 사망 위험이 오히려 증가하는 것으로 보고되었다[44]. 아울러 중성지방 수치가 가장 낮은 집단에서 나타난 결과에 대해, 중성지방 수치 저하는 영양 상태 저하, 허약, 만성 염증 상태 등 전반적인 건강상태의 취약성과 연관되었을 가능성이 있다고 언급한 바 있다[44]. 선행연구가 이미 질환을 보유한 대상자를 중심으로 수행되었다는 점에서, 일반 인구를 대상으로 한 본 연구에 직접적으로 적용하는 데에는 한계가 있을 수 있다. 그럼에도 불구하고 선행연구의 맥락을 고려할 때, 본 연구에서 관찰된 Q1 집단의 상대적으로 높은 대장암 발생 위험 역시 낮은 중성지방 자체의 영향이 아니라, 여러 요인이 복합적으로 반영되어 나타난 결과로 해석될 수 있다. 우선, Q1 집단은 현재 흡연, 현재 음주, 비만, 당뇨병 등 주요 위험요인의 노출 비율이 네 집단 중 가장 낮았음에도 불구하고, Q2 집단 보다 상대적으로 높은 대장암 발생 위험을 보였다. 이에 본 연구에서 측정되지 않았지만 기저 건강상태의 취약성이나 잠재적 만성질환 등의 요인이 대장암 발생 위험에 영향을 미쳤을 가능성이 있는 것으로 생각된다. 또한 기반조사 시점에서 측정된 중성지방 수치를 기준으로 사분위 집단을 분류하였기 때문에, 추적 기간 동안의 중성지방 변화 양상을 반영하지 못하였다는 제한점도 있다. 특히 Q1 집단은 상대적으로 연령이 낮고 여성의 비율이 높아, 시간의 경과에 따라 중성지방 수치가 증가하거나 변화했을 가능성이 있으며, 이러한 장기적인 변화가 대장암 발생 위험 추정에 영향을 미쳤을 가능성도 고려할 필요가 있다. 그럼에도 불구하고, 본 연구 결과는 중성지방 관리에서 낮은 중성지방 수치를 항상 대사적으로 건강한 상태로 해석하는 데에는 주의가 필요함을 시사하며, 향후 반복 측정 자료를 활용한 연구를 통해 이러한 비선형적 연관성을 보다 정밀하게 검증할 필요가 있다고 생각된다.

보정변수 중 다변량 콕스 비례위험 회귀모형에서 연령, 2형 당뇨병, 과거음주, 지방 에너지 섭취 비율 30% 초과가 대장암 발생 위험에 유의한 영향 요인으로 확인되었다. 먼저, 연령은 1세 증가할 때마다 대장암 발생 위험이 약 3% 증가하는 것으로 나타났는데, 이는 우리나라 국가 암등록통계에서 인구 10만 명당 대장암 발생률이 40~44세는 38.4명이었으나 85세 이상에서는 271.7명으로 급격히 증가하는 경향과 유사하다[2]. 이러한 증가 양상은 대장암이 노화 과정과 밀접하게 연관된 암으로 고령층에서 특히 높은 질병 부담을 보인다는 점을 반영한다. 2형 당뇨병 역시 대장암 발생 위험과 유의하게 관련된 요인으로 확인되었다. 당뇨병은 염증 반응과 인슐린 저항성 증가를 특징으로 하며, 이러한 대사적 변화가 대장암 발생 위험을 높이는 기전으로 제시되어 왔다[7]. 본 연구에서도 2형 당뇨병 유병자의 대장암 발병 위험이 1.85배 높게 나타났으며, 이는 대만의 대규모 코호트 연구[7]에서 당뇨병 유병자의 대장암 발생 위험이 2.46배 높게 나타난 결과와 린치증후군(Lynch syndrome) 보유자를 대상으로 한 가족 기반 연구에서 당뇨병 유병자의 대장암 발생 위험이 약 1.92배 높았던 보고[8]와 유사한 결과이다. 또한, 2형 당뇨병 유병자의 41%가 중성지방 Q4 집단에 포함되었다는 점은 당뇨병과 고중성지방혈증의 동반 가능성이 높음을 시사한다. 선행연구에서도 당뇨병 유병자 중 중성지방 150 mg/dL 이상이면서 총콜레스테롤 180 mg/dL 이상인 집단이 중성지방 150 mg/dL 미만이면서 총콜레스테롤 180 mg/dL 미만인 집단보다 대장암 발생 위험이 4.12배 높게 나타난 바 있다[7]. 이러한 결과는 대장암 예방 전략에서 2형 당뇨병 유병자를 대상으로 한 식이조절, 신체활동, 약물요법 등을 포함한 지속적인 중성지방 관리가 중요한 목표가 될 수 있음을 뒷받침한다 할 수 있다[7]. 지방 에너지 섭취비율이 30%를 초과하는 집단은 15% 미만인 집단에 비해 대장암 발생 위험이 약 4.32배 높게 나타났다. 이는 이란의 사례-대조군 연구에서 총 지방 섭취량이 가장 높은 사분위 집단(Q4)에서 가장 낮은 섭취 집단(Q1)보다 대장암 발생 위험이 1.77배 증가한 것으로 보고된 결과와 유사한 맥락이다[45]. 다만, 본 연구에서 지방 에너지 섭취비율이 30%를 초과하는 집단의 표본 수와 대장암 발생 건수가 매우 적어 95% 신뢰구간이 넓게 나타났으며, 이는 추정치의 안정성이 제한적일 가능성이 있음을 의미한다. 따라서 본 결과는 고지방섭취와 대장암 발생 위험 간의 연관 가능성을 탐색적으로 제시하는 수준으로 해석되어야 한다. 한편, 본 연구에서 과거음주자의 대장암 발생 위험이 비음주자에 비해 낮게 나타났으나, 이는 과거 음주 자체가 대장암 발생 위험을 낮추는 보호효과로 작용한 결과라고 보기는 어렵다고 사료된다. 과거음주자는 금주의 이유가 건강문제나 역할변화 등으로 개인마다 다양하며[46], 일부는 건강 개선을 위해 생활습관을 적극적으로 변화시켰을 가능성도 있을 것이다. 또한, 본 연구에서 과거음주자의 비율이 전체의 6.3%로 표본수가 적다는 점도 고려해본다면, 과거음주와 대장암 발생의 연관성은 논의의 근거로 활용할 수 있는 실증적 자료의 축적이 더 필요하다고 생각한다.

본 연구는 지역사회에 거주하는 40~69세 성인을 대상으로 장기간 추적한 코호트 자료를 활용하여 중성지방과 대장암 발생 위험 간의 연관성을 확인하였다는 점에서 의의가 있다. 주로 심혈관계질환의 위험요인으로 보고된 중성지방이 대장암 발생과도 유의하게 관련됨을 제시함으로써, 중성지방 관리의 중요성을 암 예방 영역까지 확장할 수 있는 근거를 마련하였다. 이를 통해 간호사는 지역사회 거주 성인을 대상으로 위험요인 평가와 생활습관 개선 방안을 마련하는 과정에서 중성지방 수치를 대장암 예방 관점에서 고려함으로써, 검진 연계를 중심으로 한 예방적 간호중재를 강화할 수 있다. 구체적으로, 병원, 산업체, 보건소, 국가건강검진 등의 실무영역에서 40~69세¬¬ 성인을 대상으로 한 중성지방 관리교육을 수행할 때 중성지방 수치가 대사질환뿐 아니라 대장암 발생 위험과도 연관될 수 있음을 안내함으로써 암 예방 교육으로의 연계를 도모할 수 있다. 특히, 중성지방 수치를 지속적으로 모니터링 하여, 지나치게 낮거나 높은 경우에는 대장암 발생 위험 평가에 주의깊게 반영할 필요가 있다. 간호사는 중성지방에 대한 기초간호학적 지식을 바탕으로, 대상자의 생리적 상태와 대사적 특성을 이해하고, 이를 근거로 식사패턴, 신체활동, 체중 관리, 절주 등 생활습관 개선을 중심으로 한 개별화된 간호중재를 계획할 수 있다. 또한, 중성지방 수치 이상으로 잠재적 위험군으로 분류된 대상자에게는 대장암 검진의 필요성과 국가검진 제도에 대한 정보를 제공하여 검진 이행을 촉진하는 간호중재 전략을 고려할 수 있다. 이러한 접근은 중성지방에 대한 기초간호적 지식이 임상 실무에서 건강 위험 관리와 예방적 간호중재의 기반으로 작용함을 보여준다.

본 연구의 강점으로는 대규모 일반 인구집단을 대상으로 표준화된 프로토콜을 적용한 장기 추적자료를 활용하여 대장암 발생 위험 예측의 타당성을 확보한 점을 들 수 있다. 또한 지질강하제 복용자, 악성암 진단자, 기반조사 후 1년 이내 대장암 진단자 등을 제외하여 대상자 선정에 노력을 기울였다. 다만, 본 연구 결과는 중소도시 및 농촌 지역에 거주하는 40~69세 성인을 대상으로, 기반조사 시점의 중성지방 수치와 자가보고에 의한 대장암 발생 정보를 분석한 연구라는 점에서 해석에 일정한 주의가 필요하다. 이에 본 연구의 주요 제한점은 다음과 같다. 첫째, 중성지방 500 mg/dL 이상인 대상자를 제외하였다는 점에서 초고중성지방혈증 집단에 대한 일반화에는 한계가 있다. 둘째, 연구 대상이 중소도시와 농촌 지역의 40-69세 성인이므로 한국 전체 인구로의 확대 해석에는 주의가 필요하다. 셋째, 기반조사 시점의 중성지방 수치만을 사용하여 시간 경과에 따른 수치 변화는 반영하지 못하였다. 넷째, 기반조사 시점에서 10개 암과 기반 조사 이후 1년 이내 진단된 대장암 환자는 제외하였지만 이외 다른 만성질환이나 전반적인 건강상태의 취약성을 확인하지는 못하였다. 다섯째, 대장암 진단 여부가 자가보고 자료를 통해 수집되었기 때문에, 진단여부 또는 진단 시점에 대한 오분류 바이어스(misclassification bias)의 가능성을 배제할 수 없다. 여섯째, 대장암 가족력은 자료의 제한으로 인해 주요 교란변수로 통제하지 못하였으며, 이에 따른 영향을 배제하지 못하였다. 이러한 제한점에도 불구하고 본 연구는 장기간 지역사회 기반 자료를 활용하여 대장암 발생 위험 감소 측면에서 중성지방 관리의 필요성을 제기하였다는 점에서 의의가 있다.

## 결론

본 연구는 지역사회기반 코호트의 20년간 추적조사 자료를 활용하여, 지역사회에 거주하는 40~69세 성인의 혈중 중성지방과 대장암 발생 위험 간의 연관성을 확인하였다. 연구결과, 중성지방 Q2 집단에서 대장암 발생률이 가장 낮았으며, Q1 집단과 Q4 집단에서는 Q2 집단에 비해 대장암 발생 위험이 유의하게 높게 나타났다. 이는 혈중 중성지방이 심혈관계 질환의 위험요인일 뿐 아니라 대장암 발생과도 연관된 잠재적인 대사적 표지자임을 시사한다. 또한, 중성지방 관리가 대장암 발생 위험 감소를 위한 예방 전략을 모색하는 데 활용될 수 있는 근거를 제공한다. 이에 따라, 중성지방을 대장암 발생의 잠재적 위험 요소로 인식하도록 돕는 교육과 중성지방과 연관된 위험요인의 체계적인 사정 결과를 토대로 금연, 절주, 건강체중 유지, 혈당 관리 등의 중재를 제공할 수 있을 것이다. 중성지방은 국가건강검진 항목에 포함되어 있어 주기적인 측정과 관리가 용이하므로, 이를 활용한 위험군 선별 및 지속적인 모니터링을 통해 중성지방 수치가 비정상적인 경우 대장암 검진 이행을 강화하는 방안을 고려할 수 있다. 본 연구는 우리나라 지역사회에 거주하는 40~69세 성인을 대상으로 중성지방과 대장암 발생 위험 간의 연관성에 대한 근거를 제공하였으나, 이를 근거기반 실무에 적용하기 위해서는 모집단 특성과 연구 방법을 다양화한 후속 연구의 축적이 필요하다. 향후 연구에서는 대상자의 기저 건강상태의 취약성을 면밀히 검토하고 추적 기간 동안 반복 측정된 자료를 활용하여 시간에 따른 중성지방 변화와 대장암 발생 위험과의 관계를 규명하는 종단 연구가 수행될 필요가 있다.

## Figures and Tables

**Table 1.** Demographic and Health-related Characteristics According to Triglyceride Groups Stratified by Quartile at Baseline (N = 8,371)

Characteristics	Categories	Triglyceride					χ^2^ (*p*)
		Total (n = 8,371)	Q1 (n = 2,104)	Q2 (n = 2,090)	Q3 (n = 2,095)	Q4 (n = 2,082)	
Age (years)	40~49	4,029 (48.1)	1,186 (29.4)	1,035 (25.7)	926 (23.0)	882 (21.9)	106.11
	50~59	2,183 (26.1)	489 (22.4)	536 (24.6)	556 (25.5)	602 (27.6)	(< .001)
	60~69	2,159 (25.8)	429 (19.9)	519 (24.0)	613 (28.4)	598 (27.7)	
Sex	Men	3,952 (47.2)	786 (19.8)	919 (23.3)	1,038 (26.3)	1,209 (31.6)	193.85
	Women	4,419 (52.8)	1,318 (29.8)	1,171 (26.5)	1,057 (23.9)	873 (19.8)	(< .001)
Alcohol use^†^	Never	3,838 (46.1)	1,062 (27.7)	994 (25.9)	949 (24.7)	833 (21.7)	50.51
	Former	521 (6.3)	123 (23.6)	121 (23.2)	139 (26.7)	138 (26.5)	(< .001)
	Current	3,966 (47.6)	909 (22.9)	968 (24.4)	988 (24.9)	1,101 (27.8)	
Smoking status^†^	Never	4,920 (59.3)	1,450 (29.5)	1,291 (26.2)	1,168 (23.8)	1,011 (20.5)	216.35
	Former	1,304 (15.7)	263 (20.2)	284 (21.8)	391 (29.9)	366 (28.1)	(< .001)
	Current	2,078 (25.0)	370 (17.8)	503 (24.2)	520 (25.0)	685 (33.0)	
TPA (MET)	Q1 (lowest)	2,123 (25.4)	443 (20.9)	505 (23.8)	589 (27.7)	586 (27.6)	49.09
	Q2	2,104 (25.1)	571 (27.1)	503 (23.9)	534 (25.4)	496 (23.6)	(< .001)
	Q3	2,077 (24.8)	574 (27.5)	539 (26.0)	483 (23.3)	481 (23.2)	
	Q4 (highest)	2,067 (24.7)	516 (25.0)	543 (26.3)	489 (23.6)	519 (25.1)	
Carbohydrate (% kcal)^†^	< 55	117 (1.5)	31 (26.5)	29 (24.7)	34 (29.1)	23 (19.7)	6.63
	55~65	926 (11.3)	233 (25.2)	213 (23.0)	226 (24.4)	254 (27.4)	(-.357)
	> 65	7,130 (87.2)	1,796 (25.2)	1,800 (25.2)	1,783 (25.0)	1,751 (24.6)	
Fat (% kcal)^†^	< 15	5,330 (65.2)	1,295 (24.3)	1,344 (25.2)	1,327 (24.9)	1,364 (25.6)	10.46
	15~30	2,784 (34.1)	747 (26.8)	685 (24.6)	699 (25.1)	653 (23.5)	(-.107)
	> 30	59 (0.7)	18 (30.5)	13 (22.0)	17 (28.9)	11 (18.6)	
Fiber (g)^†^	Q1 (lowest)	2,044 (25.0)	550 (26.9)	515 (25.2)	507 (24.8)	472 (23.1)	15.67
	Q2	2,043 (25.0)	522 (25.6)	503 (24.6)	513 (25.1)	505 (24.7)	(.074)
	Q3	2,043 (25.0)	513 (25.1)	532 (26.0)	506 (24.8)	492 (24.1)	
	Q4 (highest)	2,043 (25.0)	475 (23.3)	492 (24.1)	517 (25.3)	559 (27.3)	
Type 2 DM	No	7,305 (87.3)	1,969 (27.0)	1,893 (25.9)	1,805 (24.7)	1,638 (22.4)	236.76
	Yes	1,066 (12.7)	135 (12.6)	197 (18.5)	290 (27.1)	444 (41.8)	(< .001)
Hypertension	No	5,310 (63.4)	1,579 (29.7)	1,435 (27.0)	1,230 (23.2)	1,066 (20.1)	301.42
	Yes	3,061 (36.6)	525 (17.1)	655 (21.4)	865 (28.3)	1,016 (33.2)	(< .001)
BMI (kg/m^2^)^†^	≤ 22.9	2,584 (30.9)	968 (37.5)	724 (28.0)	548 (21.2)	344 (13.3)	570.12
	23~24.9	2,195 (26.2)	544 (24.8)	588 (26.7)	528 (24.1)	535 (24.4)	(< .001)
	≥ 25	3,590 (42.9)	591 (16.5)	778 (21.7)	1,019 (28.4)	1,202 (33.4)	
HOMA-IR^†^	< 2	6,030 (74.3)	1,728 (28.7)	1,609 (26.7)	1,468 (24.3)	1,225 (20.3)	289.41
	≥ 2 to < 2.5	1,046 (12.9)	206 (19.7)	221 (22.1)	289 (27.7)	330 (31.5)	(< .001)
	≥ 2.5	1,042 (12.8)	135 (13.0)	216 (20.7)	279 (26.8)	412 (39.5)	
HDL-C	Q1(lowest)	2,362 (28.2)	217 (9.2)	422(17.9)	686 (29.0)	1,037 (43.9)	1406.11
(mg/dL)	Q2	2,290 (27.4)	439 (19.2)	578(25.2)	668 (29.2)	605 (26.4)	(< .001)
	Q3	1,738 (20.7)	544(31.3)	524(30.1)	401 (23.1)	269 (15.5)	
	Q4 (highest)	1,981 (23.7)	904(45.6)	566(28.6)	340 (17.2)	171 (8.6)	
Total	Q1 (lowest)	2,139 (25.6)	790(36.9)	583(27.3)	438 (20.5)	328 (15.3)	438.17
cholesterol	Q2	2,059 (24.6)	547(26.6)	544(26.4)	515 (25.0)	453 (22.0)	(< .001)
(mg/dL)	Q3	2,112 (25.2)	446(21.1)	528(25.1)	571 (27.0)	567 (26.8)	
	Q4 (highest)	2,061 (24.6)	321(15.6)	435(21.1)	571 (27.7)	734 (35.6)	

Values are presented as n (%). Q = Quartile; TPA = Total physical activity; MET = Metabolic equivalent of task; DM = Diabetes mellitus; BMI = Body mass index; HOMA-IR = Homeostatic model assessment of insulin resistance index; HDL-C = High-density lipoprotein cholesterol. ^†^Missing value.

**Table 2.** Association Between Demographic and Health-related Characteristics and Colorectal Cancer Incidence (N = 8,371)

Characteristics	Categories	Number at risk	CRC event (%)	HR	95% CI	*p*
Age (years)	40~49	4,029	43 (1.07)	1		
	50~59	2,183	36 (1.65)	1.56	1.00~2.43	.050
	60~69	2,159	25 (1.16)	1.37	0.84~2.24	.214
Sex	Men	3,952	60 (1.52)	1		
	Women	4,419	44 (1.00)	0.61	0.42~0.91	.014
Alcohol use^†^	Never	3,838	47 (1.22)	1		
	Former	521	2 (0.38)	0.34	0.08~1.39	.131
	Current	3,966	55 (1.39)	1.16	0.78~1.74	.470
Smoking status^†^	Never	4,920	48 (0.98)	1		
	Former	1,304	23 (1.76)	1.89	1.15~3.11	.012
	Current	2,078	32 (1.54)	1.75	1.12~2.74	.014
TPA (MET)	Q1(lowest)	2,123	26 (1.22)	1		
	Q2	2,104	25 (1.19)	0.95	0.55~1.64	.842
	Q3	2,077	27 (1.30)	1.02	0.60~1.75	.944
	Q4 (highest)	2,067	26 (1.26)	0.95	0.55~1.63	.850
Carbohydrate (% kcal)^†^	< 55	117	3 (2.56)	1		
	55~65	926	12 (1.30)	0.50	0.14~1.78	.287
	> 65	7,130	88 (1.23)	0.47	0.15~1.50	.203
Fat (% kcal)^†^	< 15	5,330	65 (1.22)	1		
	15~30	2,784	35 (1.26)	1.03	0.68~1.55	.888
	> 30	59	3 (5.08)	4.11	1.29~13.06	.016
Fiber (g)^†^	Q1 (lowest)	2,044	20 (0.98)	1		
	Q2	2,043	30 (1.47)	1.50	0.85~2.64	.160
	Q3	2,043	31 (1.52)	1.61	0.92~2.83	.096
	Q4 (highest)	2,043	22 (1.08)	1.11	0.61~2.04	.073
Type 2 DM	No	7,305	85 (1.16)	1		
	Yes	1,066	19 (1.78)	1.81	1.10~2.97	.020
Hypertension	No	5,310	70 (1.32)	1		
	Yes	3,061	34 (1.11)	0.90	0.60~1.36	.627
BMI (kg/m^2^)^†^	≤ 22.9	2,584	35 (1.35)	1		
	23~24.9	2,195	30 (1.37)	0.98	0.60~1.59	.930
	≥ 25	3,590	39 (1.09)	0.77	0.52~1.15	.251
HOMA-IR^†^	< 2	6,030	71 (1.18)	1		
	≥ 2 to < 2.5	1,046	15 (1.43)	1.24	0.71~2.16	.455
	≥ 2.5	1,042	16 (1.54)	1.35	0.79~2.32	.278
HDL-C (mg/dL)	Q1 (lowest)	2,362	27 (1.14)	1.05	0.60~1.86	.859
	Q2	2,290	32 (1.40)	1.28	0.74~2.22	.377
	Q3	1,738	24 (1.38)	1.26	0.70~2.26	.444
	Q4 (highest)	1,981	21 (1.06)	1		
Total cholesterol	Q1 (lowest)	2,139	26 (1.22)	1		
(mg/dL)	Q2	2,059	29 (1.41)	1.12	0.66~1.91	.665
	Q3	2,112	19 (0.9)	0.75	0.41~1.35	.335
	Q4 (highest)	2,061	30 (1.46)	1.24	0.73~2.09	.426

CRC = Colorectal cancer; HR = Hazard ratio; CI = Confidence interval; Q = Quartile; TPA = Total physical activity; MET = Metabolic equivalent of task; DM = Diabetes mellitus; BMI = Body mass index; HDL-C = High-density lipoprotein cholesterol; HOMA-IR = Homeostatic model assessment of insulin resistance index. ^†^Missing value.

**Table 3.** Hazard Ratios for the Incidence of Colorectal Cancer According to Triglyceride Groups Stratified by Quartile (N = 8,371)

Characteristics	CRC events (%)	Person-years		Incidence rate per 1,000 person-years	Crude model		Model I		Model II	
		Median	Sum		HR (95% CI)	*p*	HR (95% CI)	*p*	HR (95% CI)	*p*
Total	104 (1.24)	19.50	132,624	0.78						
Triglyceride, Q2	16 (0.77)	19.50	33,349	0.48	1		1		1	
Q1 (lowest)	32 (1.52)	19.50	33,843	0.95	1.97 (1.08~3.60)	.026	2.12 (1.16~3.87)	.014	2.13 (1.15~3.95)	.016
Q3	23 (1.10)	19.41	32,736	0.70	1.48 (0.78~2.81)	.226	1.43 (0.75~2.70)	.274	1.27 (0.65~2.48)	.487
Q4 (highest)	33 (1.59)	19.41	32,696	1.01	2.13 (1.17~3.87)	.013	1.96 (1.08~3.56)	.028	1.95 (1.02~3.73)	.043
Age (years)^†^							1.02 (1.00~1.04)	.091	1.03 (1.00~1.05)	.027
Sex, men & women							0.60 (0.40~0.89)	.012	0.76 (0.39~1.50)	.431
Alcohol, never & former									0.11 (0.02~0.83)	.032
never & current									0.85 (0.52~1.39)	.522
Smoking, never & former									1.95 (0.94~4.07)	.074
never & current									1.69 (0.84~3.38)	.142
TPA, Q1 & Q2									1.03 (0.58~1.82)	.923
Q1 & Q3									1.05 (0.60~1.85)	.864
Q1 & Q4									0.94 (0.53~1.67)	.829
BMI (kg/m^2^), ≤ 22.9 & 23~24.9									0.98 (0.59~1.65)	.962
≤ 22.9 & ≥ 25									0.71 (0.42~1.20)	.198
Fat (% kcal), < 15 & 15~30									0.98 (0.63~1.52)	.929
< 15 & > 30									4.32 (1.33~14.06)	.015
Type 2 DM, no & yes									1.85 (1.04~3.29)	.036
Hypertension, no & yes									1.34 (0.47~1.17)	.198
HOMA-IR, < 2 & ≥ 2 to < 2.5									1.24 (0.69~2.23)	.478
< 2 & ≥ 2.5									1.25 (0.67~2.32)	.470
HDL-cholesterol^†^									1.00 (0.98~1.02)	.977
Total cholesterol^†^									1.00 (0.99~1.01)	.779

CRC = Colorectal cancer; HR = Hazard ratio; CI = Confidence interval; Q = Quartile; TPA = Total physical activity; BMI = Body mass index; DM = Diabetes mellitus; HOMA-IR = Homeostatic model assessment of insulin resistance index; HDL = High-density lipoprotein. ^†^Age, HDL-cholesterol, and total cholesterol were analyzed as continuous variables.

## Data Availability

The data that support the findings of this study are available from the Clinical & Omics Data Archive (CODA) (https://coda.nih.go.kr/frt/index.do). Access to the dataset is available to qualified researchers who obtain approval from the CODA Data Access Committee for approved research purposes.
